# Untargeted metabolomics fingerprints in seminal plasma of patients with abnormal sperm morphology using high-performance liquid chromatography and mass spectrometry

**DOI:** 10.3389/fmolb.2025.1578998

**Published:** 2025-06-17

**Authors:** Serena Correnti, Giuseppina Fanelli, Mariaimmacolata Preianò, Veronica Lelli, Mariagrazia Tarantino, Annalisa Fregola, Massimo Bitonti, Emanuela Chiarella, Anna Maria Timperio, Sara Rinalducci, Rocco Savino, Rosa Terracciano

**Affiliations:** ^1^ Department of Health Sciences, Magna Græcia University, Catanzaro, Italy; ^2^ Department of Ecological and Biological Sciences (DEB), University of Tuscia, Viterbo, Italy; ^3^ Department of Experimental and Clinical Medicine, Magna Græcia University, Catanzaro, Italy; ^4^ Urogyn Biotech S. R. L., Catanzaro, Italy; ^5^ Department of Medical and Surgical Sciences, Magna Græcia University, Catanzaro, Italy

**Keywords:** male infertility, human seminal plasma, teratozoospermia, mass spectrometry, metabolomics, biomarkers, reproductive medicine, precision medicine

## Abstract

Teratozoospermia, a qualitative sperm disorder characterized by abnormal sperm morphology, represents one of the causes of male infertility worldwide. The metabolic analysis of human seminal plasma (SP), can provide insights into the underlying molecular mechanisms of this condition, identifying novel biomarkers and facilitating the development of diagnostic tests. In this study, an untargeted High-Performance Liquid Chromatography-Mass Spectrometry (HPLC-MS) approach was performed to explore SP metabolic alterations associated with teratozoospermia. SP samples from 15 teratozoospermic (TZ) vs. 20 normozoospermic (NZ) subjects were analyzed to identify metabolic pathways linked to sperm morphology dysfunction. Multivariate statistical analysis, including Partial Least Squares Discriminant Analysis (PLS-DA) and Orthogonal PLS-DA, revealed a distinct separation between TZ and NZ, highlighting 14 significantly altered metabolites. Based on Variable Importance in Projection scores, O-acetyl-L-serine showed the highest score. Main findings include alterations in Creatine, Histidine, Adenine, Allantoin and Deoxyuridine levels, suggesting perturbations in inflammation, oxidative stress and sperm DNA damage in teratozoospermia. Correlation and Receiver Operating Characteristic (ROC) analyses identified potential biomarkers, including O-acetyl-L-serine, Creatine, and Histidine, with robust discriminatory power (AUC >0.7). These findings highlight potential metabolic pathways implicated in the pathophysiology of teratozoospermia and provide a foundation for enabling personalized patient management with precision treatment.

## 1 Introduction

Infertility is a rising global health issue, affecting around 15% of reproductive-age couples in the world (Ferlin and Foresta, 2020; Agarwal et al., 2021), with significant psychological, social, and economic repercussions.

Among sperm abnormalities, teratozoospermia represents a qualitative dysfunction of sperm that affects individuals worldwide (Das et al., 2022), characterized by the presence of morphologically abnormal spermatozoa in semen. The abnormal sperm cell’s morphology results from defective cell differentiation during spermatogenesis, associated with several genetic and environmental factors and lifestyle habits (Candela et al., 2021; Agarwal et al., 2014). Teratozoospermia has been associated not only with lower semen quality but also with increased DNA fragmentation, severe inflammatory status and excessive production of reactive oxygen species (ROS), which are indicators of sperm damage (Agarwal et al., 2014; Ammar et al., 2020). According to the World Health Organization (WHO) 2021 guidelines, this condition is defined by a percentage of normal spermatozoa below the lower reference limit of 4% (World Health Organization, 2021). Typically, a morphologically normal spermatozoa have specific features, including an oval-shaped head (2.5–3.5 μm in width and 5–6 μm in length), a normal acrosome, a midpiece about 4.0–5.0 μm long, and a tail approximately 50 μm in length (De Braekeleer et al., 2015). Teratozoospermia includes a diverse range of abnormal sperm phenotypes, which can impact various parts of the sperm, such as the head, neck, midpiece, and tail (Kyrgiafini et al., 2023).

The prevalence of teratozoospermia varies significantly across different populations. A large-scale study conducted in Qatar on 13,892 infertile men reported a prevalence of 48.7% teratozoospermic men, highlighting notable geographical differences. For instance, patients from the Middle East and North Africa (MENA) region exhibited lower semen quality and a higher incidence of teratozoospermia compared to non-MENA regions (South Asia, East Asia and Pacific, Europe and Central Asia, Sub − Saharan Africa, Latin America and the Caribbean, and North America) (Elbardisi et al., 2018). In addition the study by Owolabi et al. (2013) in Nigeria reported an incidence of 18.5% for teratozzospermia in a group of 661 male partners of infertile couples, while Morey-León et al. (2020) in Ecuador found a higher prevalence of 27.9% % in 204 semen samples from patients with fertility disorders aged 20–57 years, with an additional 8.8% of cases classified as oligoteratozoospermia. In particular the study reported a higher prevalence of teratozoospermia among individuals aged 30 to 39, suggesting a possible age-related decline in sperm quality (Morey-León et al., 2020).

Sperm morphology has been considered a predictor of natural conception and fertilization success (Atmoko et al., 2024), and the presence of abnormal spermatozoa has been linked to reduced pregnancy rates, and fetal DNA damage (Cao et al., 2017).

However, in most cases, the exact causes and origins of morphological sperm dysfunctions are far from well understood, although some notable associations have been reported in previous studies (De Braekeleer et al., 2015; Danis and Samplaski, 2019; Franken, 2015; Linn et al., 2021). Therefore, a deeper understanding of the mechanisms behind these dysfunctions is crucial for comprehending their impact on conception rates and reproductive outcomes, as well as for developing more effective treatment strategies.

In this regard, investigating new and reliable molecular biomarkers using omics technologies is highly valuable for gaining insights into the molecular aspects of both normal and pathological conditions.

Metabolomics, in particular, has emerged as a comprehensive approach to identify specific biomarkers linked to reproductive issues (Aiel et al., 2016; Aderemi et al., 2021; Panner Selvam et al., 2021; Correnti et al., 2023). This method not only captures the final products of gene and protein expression but also reflects the cellular signaling processes arising from interactions between the genome/proteome and environmental factors, bringing it closer to the actual phenotype (Guijas et al., 2018; Blaurock et al., 2022). Identifying unique metabolites in biological samples from infertile men offers significant potential for enhancing our understanding of male infertility and its underlying causes.

Among various biological samples, seminal plasma (SP)—the fluid portion of semen—has proven to be a promising, accessible, and convenient matrix for metabolomic analysis in evaluating and diagnosing male infertility (Blaurock et al., 2022; Preianò et al., 2023). Identifying and studying biomarkers in SP could have far-reaching implications not only for diagnosing but also for potentially treating male infertility.

Up to date, the SP metabolomics of teratozoospermia has been reported in a very limited number of investigations, which yet need to be validated (Mehrparvar et al., 2020; Hosseini et al., 2023). Despite advancements in metabolomics, the biochemical pathways in SP which can be linked to teratozoospermia remain poorly understood.

Hence, a better understanding of the molecular and pathophysiological mechanisms associated with teratozoospermia could be achieved by identifying metabolic alterations in SP from teratozoospermic (TZ) men. Such knowledge may contribute to improving the characterization of this condition and potentially aid in refining diagnostic approaches. Here, we performed for the first time an untargeted high-pressure liquid chromatography (HPLC)-MS based metabolomics approach in SP samples from TZ and normozoospermic (NZ) men to identify metabolic signatures linked to abnormal sperm morphology. This approach may help elucidate the metabolic pathways associated with teratozoospermia and improve our understanding of the underlying mechanisms contributing to male infertility.

## 2 Materials and methods

### 2.1 Patient enrollment

This study was approved by the Declaration of Helsinki, upon approval by the Ethics Committee of MAGNA GRAECIA UNIVERSITY and MATER DOMINI HOSPITAL (protocol code 2014.39, date of approval 16 April 2014) and included 35 subjects (15 infertile patients diagnosed as TZ and 20 fertile NZ men as control group). All individuals signed informed written consent and provided a questionnaire to obtain information on age, smoking habits, alcohol use and use or abuse of other substances and drugs (Supplementary Table S1). Subjects with vasectomy, orchitis, testicular trauma, sexually transmitted disease, varicocele, inguinal hernia operation, or cryptorchism were excluded from the study. Semen samples of the recruited subjects were acquired through masturbation into sterile containers, following a period of 3–5 days of sexual abstinence. Anonymously, all samples were processed and analyzed. A part of each semen sample was analyzed for routine evaluation of semen parameters such as sperm concentration, pH, normal sperm morphology and motility. These analyses were carried out by standard procedures according to the 5th WHO laboratory manual for the examination and processing of human semen (World Health Organization, 2021). Only infertile patients with abnormal sperm morphology (<4%) and normal sperm motility, sperm count and volume were included in the present study. All characteristics of enrolled subjects are summarized in Table 1.

**TABLE 1:** Clinical characteristics of the subjects.

Patients’s characteristics (mean ± SD)		Normozoospermic (NZ)	Teratozoospermic (TZ)
Demographic data	Number of subjects	20	15
	Age (years)	26.3 ± 6.5	33.7* ± 7.6
Semen parameters	Ejaculated volume (mL)	3.9 ± 2	3.7 ± 2.3
	pH	7.6 ± 0.3	7.6 ± 0.4
	Sperm count (million)	205.3 ± 134	172.1 ± 51.5
	Progressive motility (%)	47.7 ± 8.3	41.6* ± 7.9
	Total motility (%)	58.9 ± 9.3	56.3 ± 7
	Morphology (%)	6.5 ± 2.5	2.2** ± 1

Data are expressed as numbers or mean ± standard deviation.*p-value <0.05; **p-value <0.000001.

### 2.2 Preparation of SP

Semen samples were processed as described in previous studies (Correnti et al., 2023; Correnti et al., 2022). Briefly, each ejaculate was allowed to liquefy for 15 min at 37°C. Semen parameters were assessed according to guidelines provided by the WHO in 2021. After the complete liquefaction, a protease inhibitor cocktail (PIC) was added to each liquefied sample in 1:100 v/v ratio. The aliquots were processed to obtain SP. In particular, each clinical sample was centrifuged at 15,000 × g for 15 min at 4°C, to ensure complete separation of cell debris and spermatozoa from the supernatant, representing the SP (Wu et al., 2019; Herwig et al., 2013; Pilch and Mann, 2006; Turunen et al., 2022; Drabovich et al., 2011) The supernatant was collected, and an aliquot was microscopically assessed to confirm the absence of spermatozoa. The SP samples were then aliquoted and stored at −80°C until use.

### 2.3 Metabolites extraction procedure

Metabolites extraction was performed according to the following procedure. Aliquots of 100 µL of each SP sample were treated with a cold (−20°C) solution of 60% methanol/40% chloroform. The tubes were vortexed for 30 s (5 times) and transferred to −20°C overnight. The tubes were subsequently centrifuged at 135,00 × g for 10 min at 4°C, supernatants were recovered and dried for 2 h to obtain visible pellets. Finally, the dried samples were re-suspended in 100 µL water containing 0.1% formic acid and transferred to glass autosampler vials for LC/MS analysis.

### 2.4 HPLC-MS analysis

20 µL of extracted supernatant samples were injected into a HPLC system (Ultimate 3,000, Thermo Fisher Scientific, Waltham, MA, United States) and were run in positive mode: samples were loaded onto a Reprosil C18 column (2.0 mm × 150 mm, 2.5 μm—Dr. Maisch, Ammerbuch, Germany) for metabolite separation. Chromatographic separations were achieved at a column temperature of 30°C and a flow rate of 0.2 mL/min. For positive ion mode (+) MS analyses, a 0%–60% linear gradient of solvent A (ddH2O with 0.1% formic acid) to B (acetonitrile with 0.1% formic acid) was employed over 20 min, returning to 100% A in 2 min and a 6 min post-time solvent A hold. Acetonitrile, formic acid and HPLC-grade water and standards (≥98% chemical purity) were purchased from Sigma Aldrich. The HPLC system was coupled online with a mass spectrometer Q Exactive (Thermo Fisher Scientific, United States) scanning in full MS mode (2 μscans) at 70,000 resolution in the 67–1,000 m/z range, with a target of 1e6 ions, maximum ion injection time (IT) of 35 ms, 3.8 kV spray voltage, 40 sheath gas and 25 auxiliary gas (Fanelli et al., 2023). Raw files of replicates were exported, converted into mzXML format using MassMatrix (Cleveland, OH, United States), and processed with MAVEN 8.1 software (http://maven.princeton.edu/, accessed on 4 April 2023). Mass spectrometry chromatograms were analyzed for peak alignment, matching, and comparison of parent and fragment ions, as well as tentative metabolite identification, within a 2 ppm mass-deviation range between observed and expected results against the imported KEGG database. Metabolite assignments were performed with filtering applied based on predefined parameters, ensuring the selection of high-confidence metabolites. Additionally, manual peak curation was available when necessary to refine metabolite identification and exclude potential contaminants. The list of all the metabolites used in the analysis is reported in Supplementary Table S2.

### 2.5 Statistical analysis

After testing for data normal distribution, a two-sided Student’s t-test on peak intensity was performed, with the level of statistical significance set to p-values of <0.05. Metabolomic data were normalized in MetaboAnalyst (6.0). Furthermore, a comprehensive suite of univariate analyses, including T-tests, fold change analysis, and volcano plots, was used. Additionally, Partial Least Squares Discriminant Analysis (PLS-DA) and Orthogonal Partial Least Squares Discriminant Analysis (OPLS-DA) were accomplished by MetaboAnalyst (6.0) to identify the specific group of metabolites responsible for the overall discrimination ability. Box plot analysis was also performed by OriginLab® software (version 7.0, OriginLab Corporation, Northampton, MA, United States) and correlation analysis (Spearman) was carried out in JASP open-source statistical software (Version 0.18.1, https://jasp-stats.org). This analysis was used to provide valuable insights into the clinical relevance and the potential diagnostic significance of the identified metabolites. Finally, Receiver Operating Characteristic (ROC) curve and cumulative ROC curve analysis were performed using MetaboAnalyst (6.0), to establish the diagnostic performance of biomarkers and evaluate their ability to discriminate between different groups or conditions. Pathway analysis, performed by MetaboAnalyst (6.0), was used to detect significantly altered metabolic pathways in teratozoospermia compared to the control group.

## 3 Results

### 3.1 Study participants

The study included 35 subjects, with 20 men having a normal spermiogram (fertile) and 15 infertile patients with an alteration of sperm morphology (TZ). Table 1 summarizes the clinical and semen characteristics of study participants. There was no difference in sperm concentration, pH, ejaculate volume and total sperm motility between the two groups. Interestingly, a very highly statistically significant p-value was obtained for sperm morphology (p-value <0.000001). In particular, this semen parameter was decreased in teratozoospermia vs. normozoospermia, as expected according to patient classification.

Additional differences between fertile men and patients with teratozoospermia were statistically significant in terms of age and progressive motility (p-value <0.05). Compared to controls, TZ men showed a higher average age and lower progressive motility (%) (Table 1).

### 3.2 Revealing metabolic differences between NZ fertile and TZ men by untargeted HPLC-MS analysis

**FIGURE 1:**
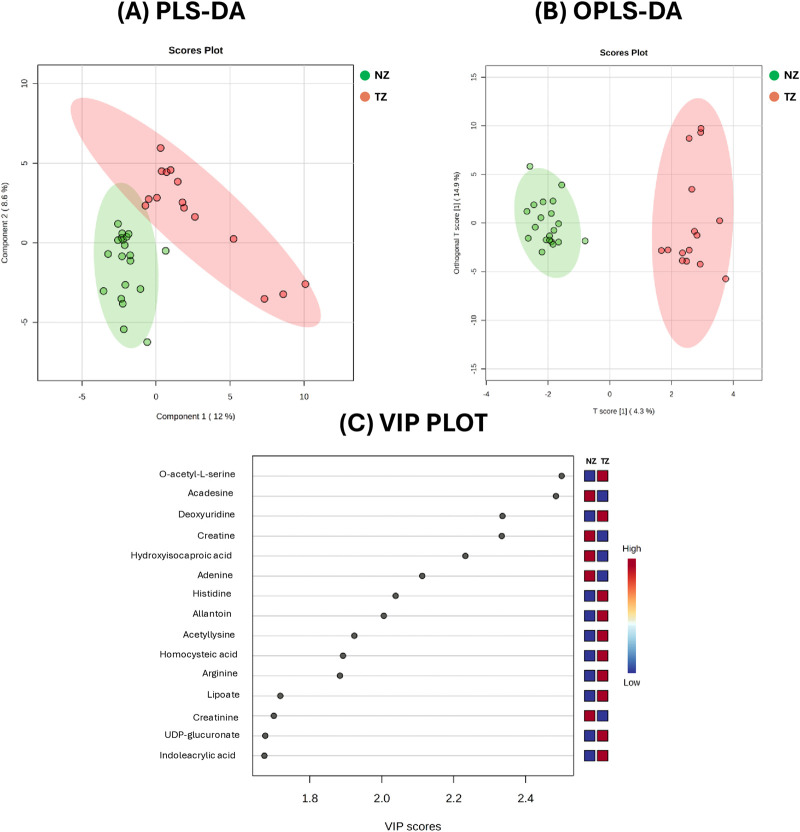
Metabolic analysis of Normozoospermic (NZ) and Teratozoospermic (TZ) patients using multivariate statistical methods. **(A)** The score plot from Partial Least Squares Discriminant Analysis (PLS-DA) and **(B)** The score plot from Orthogonal Partial Least Squares Discriminant Analysis (OPLS-DA), acquired by Metaboanalyst software. **(C)** The VIP score plot from PLS-DA of the top 15 metabolites compounds that differ between NZ and TZ patients. The colored boxes on the right indicate the relative concentrations of these compounds, with blue representing lower concentrations and red representing higher concentrations.

SP samples from TZ patients and NZ controls were first analyzed using HPLC-MS and metabolomics data were compared between the two analyzed groups, using statistical data analysis methods, in order to identify new sensitive biomarkers of the specific subfertility condition, as defined by semen analysis. To characterize metabolic differences, multivariate statistical analysis via PLS-DA and OPLS-DA was performed. These models visually discriminated between NZ (fertile) and TZ groups, highlighting metabolic variations between them (Figure 1).

The score plots from PLS-DA (Panel A) and OPLS-DA (Panel B), comparing the metabolite profiles of 20 normozoospermic (green) and 15 teratozoospermic (red) men, displayed separation between the two groups. Specifically, all TZ SP samples were clustered on the right, while NZ SP samples were clustered on the left of the plots (Figures 1A,B). The explained variance (R^2^ = 0.92, Q^2^ = 0.51) was greater than 0.5, indicating a model with a reasonable fit with good predictive power suggesting that while some metabolic differences exist, they account for only a moderate portion of the overall variance.

The variable importance in the projection (VIP) plot from the PLS-DA, reported in Figure 1C, summarizes the most important metabolites (top 15) in discriminating the two clinical groups. In particular, variables with a VIP >1 were interpreted as highly influential for group segregation and selected for subsequent analysis. A larger VIP value indicated a more significant contribution to the separation between groups. O-acetyl-L-serine, acadesine, deoxyuridine, creatine, hydroxyisocaproic acid, adenine and histidine were among the top 15 metabolites considered relevant for group dis-crimination (Figure 1C).

**FIGURE 2:**
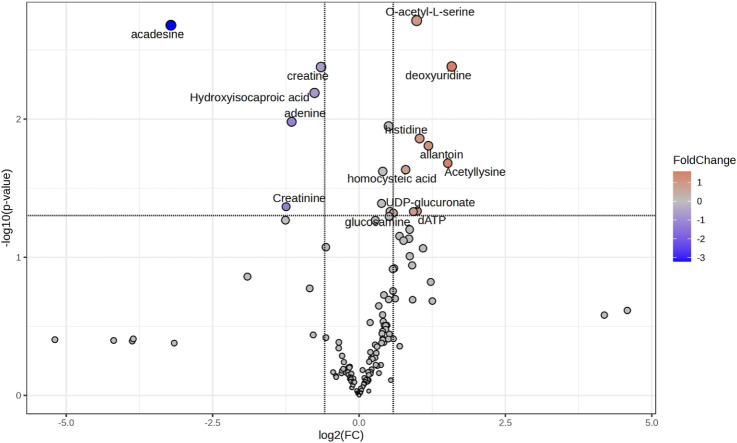
Volcano plot showing the most discriminant metabolites allowing the distinction of seminal plasma (SP) samples from NZ or TZ patients. The statistical significance was set at p-value <0.05 and Fold Change >1.5. Blue or red dots indicate, respectively, significantly decreased or increased metabolites concentrations, in TZ vs. NZ subjects. Gray dots represent compounds that did not show significant differences. Volcano plot was generated by using the Metaboanalyst software.

Volcano plot analysis (Figure 2) identified differential metabolites using fold-change (FC) and t-test analysis, with log2 (fold-change >1.5) plotted on the X-axis and -log10 (p-value) from the t-test on the Y-axis. The more distant a metabolite’s position is from the origin (0, 0) on the chart, the more relevant the feature becomes. The comparison between TZ samples and controls NZ revealed 14 metabolites with statistically significant differences. Of these, 5 were decreased (blue dots on the chart), while 9 were increased (red dots) in TZ men vs. NZ (Figure 2).

Table 2 lists all the 14 differentially expressed metabolites between the two groups that resulted from the combination of VIP plot and Volcano plot analysis, setting the criteria as VIP score >1, FC > 1.5 and p-value (Student’s t-test) < 0.05. These metabolites were selected as the major compounds responsible for the differentiation between NZ fertile and TZ infertile groups.

**TABLE 2:** List of altered metabolites between NZ and TZ patients, that resulted from the combination of VIP plot analysis (VIP score >1) and Volcano plot analysis (FC > 1.5 and p-value <0.05).

Metabolite	VIP-score	Fold change^a^	*p*-value	Metabolic pathway	Class
O-acetyl-L-serine	2.5	1.9782	0.001942	Cysteine and methionine metabolism	Amino acid
Acadesine	2.4842	0.1078	0.002097	-	Ribonucleosides
Deoxyuridine	2.3354	2.9957	0.004165	Pyrimidine metabolism	Pyrimidine nucleosides
Creatine	2.3336	0.63841	0.004198	Arginine and Proline metabolism Glycine, Serine and Threonine metabolism	Carboxylic acid and derivates
Hydroxyisocaproic acid	2.2325	0.59076	0.006469	-	Fatty acyls
Adenine	2.1124	0.4505	0.010462	Purine metabolism	Imidazolpyrimidines
Histidine	2.0386	2.0484	0.013835	β-Alanine metabolism Histidine metabolism	Amino acid
Allantoin	2.0057	2.2737	0.015606	Purine metabolism	Azoles
Acetyllysine	1.9237	2.8603	0.02087	-	Amino acid
Homocysteic acid	1.8921	1.7365	0.02326	Neuroactive ligand-receptor interaction	Amino acid
Creatinine	1.6999	0.42201	0.043127	Arginine and proline metabolism	Carboxylic acid and derivates
UDP-glucuronate	1.676	1.9941	0.046344	Amino sugar and nucleotide sugar metabolism	Lactones
dATP	1.6728	1.9027	0.046796	Purine metabolism	Purine nucleotides
Glucosamine	1.6645	1.501	0.047966	Amino sugar and nucleotide sugar metabolism	Organic compound

^a^ Fold change value refers to the “Teratozoospermic versus Normozoospermic” change values.

**FIGURE 3:**
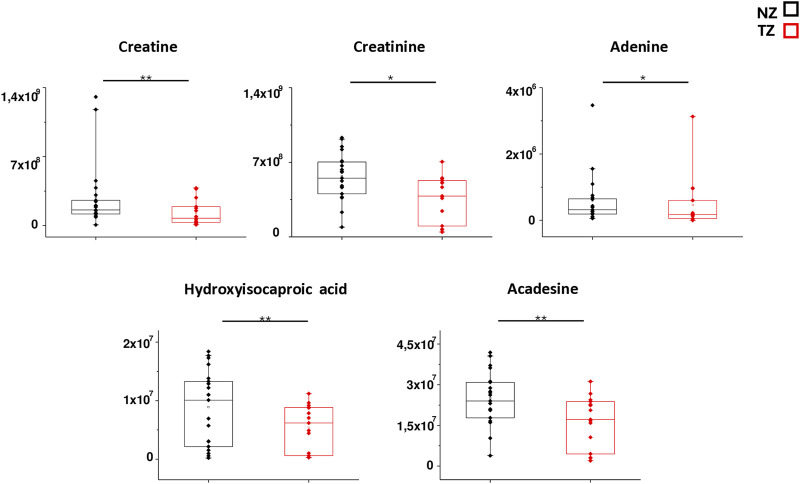
Box plot analysis of main discriminant compounds. Box plot of the peak intensities for the 5 statistically reduced metabolites in TZ patients compared to NZ. The *p*-values were calculated by Student’s t-test and the asterisks show the level of significance between the two groups.* *p*-values < 0.05,** *p*-values < 0.01.

**FIGURE 4:**
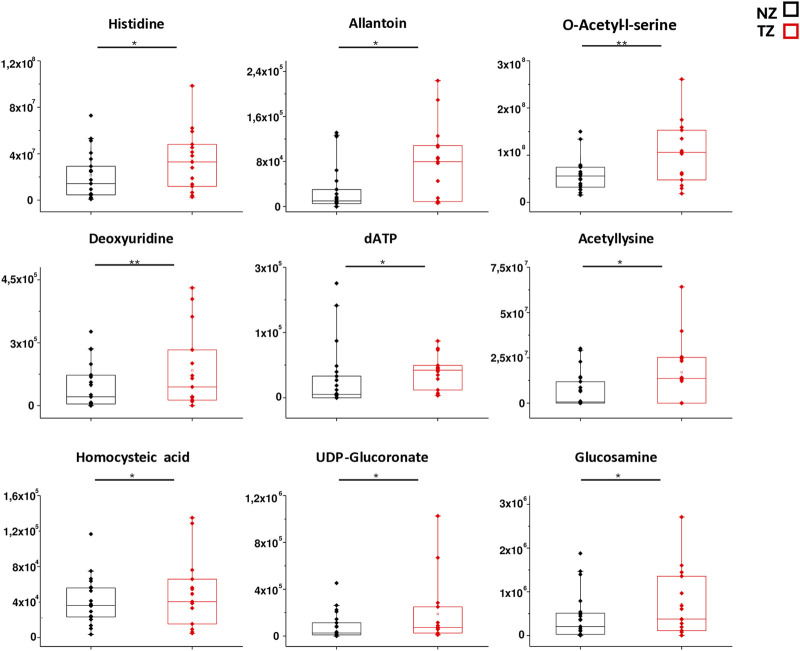
Box plot analysis of main discriminant compounds. Box plot of the peak intensities for the 9 statistically increased metabolites compounds in TZ patients compared to NZ. The *p*-values were calculated by Student’s t-test and the asterisks show the level of significance between the two groups.* *p*-values < 0.05,** *p*-values < 0.01.

In order to better mark differences in metabolites levels, the graphical comparison of peak intensities (autoscale normalized, i.e., mean-centered and divided by the standard deviation of each variable) illustrated in the box plots was reported in Figures 3, 4. Figure 3 shows that significantly reduced metabolites in TZ included Acadesine, Creatine, Hydroxyisocaproic acid, Adenine and Creatinine. On the other hand, significantly elevated metabolites were O-acetyl-L-serine, Deoxyuridine, Histidine, Allantoin, Acetyllysine, Homocysteic acid, UDP-glucuronate, dATP and Glucosamine (Figure 4).

### 3.3 Evaluating the potential clinical relevance of the altered metabolites as biomarkers of teratozoospermia

**FIGURE 5:**
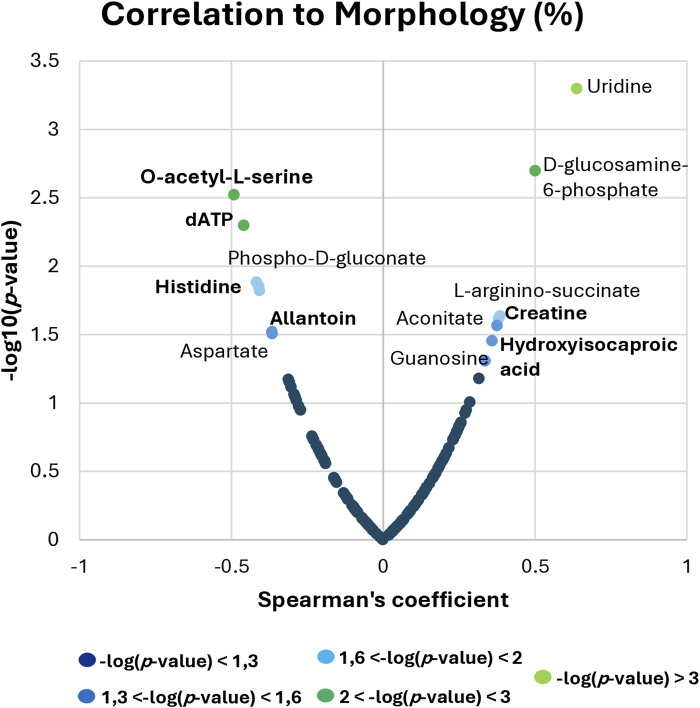
Spearman correlation analysis between metabolites and sperm morphology. The x-axis shows the Spearman’s correlation coefficient, while the y-axis displays the significance of the correlation (−log10 of the *p*-value).

**FIGURE 6:**
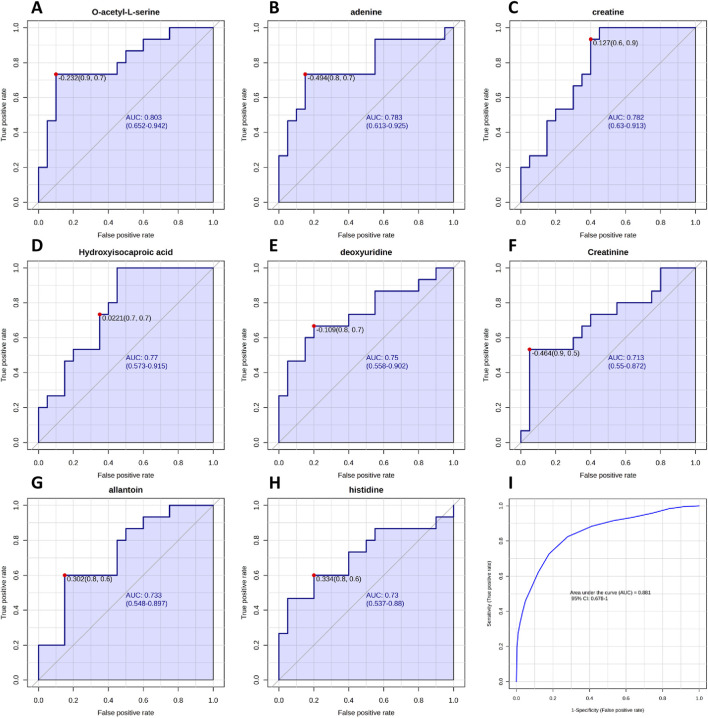
Receiver Operating Characteristic (ROC) curves analysis of metabolites between NZ men and TZ patients, based on area under the curve (AUC). **(A–H)** ROC curves constructed for metabolites that exhibited the most effective diagnostic performance and ability to distinguish between the two groups. **(I)** Cumulative ROC curve constructed by combining all metabolites that showed good/moderate diagnostic capability. ROC and cumulative ROC curves were acquired by Metaboanalyst software.

After obtaining a successful discrimination between fertile and TZ patients and having identified different metabolites that contributed to this differentiation, the evaluation of the potential role of these metabolites as biomarkers of teratozoospermia was performed, through correlation analysis and ROC curve analysis (Figures 5, 6). Since teratozoospermia involves an alteration in sperm morphology, we conducted a correlation analysis (Spearman) between metabolites and the morphology parameter, in order to evaluate specific relationship of metabolites with teratozoospermia (Figure 5).

By setting the criteria to p-value <0.05, different metabolites were found to correlate with sperm morphology. Among the most relevant correlations, Uridine, D-glucosamine-6-phosphate, L-arginino-succinate, Aconitate, Guanosine, Creatine and Hydroxyisocaproic acid were positively correlated, while O-acetyl-L-serine, dATP, Phospho-D-gluconate, Histidine, Allantoin and Aspartate showed negative correlation with sperm morphology (Figure 5).

Subsequently, altered metabolites were further analyzed by ROC curve analysis in order to understand if they could be considered candidate biomarkers of teratozoospermia in terms of diagnostic sensitivity and specificity. By plotting the true positive rate (sensitivity) against the false positive rate (1-specificity), the ROC curve provides information about the diagnostic ability of the biomarker to discriminate between two diagnostic groups, based on the evaluation of the area under the curve (AUC). The closer the AUC is to 1, the better the biomarker is at correctly differentiating the groups. Our results are shown in Figure 6. Among all identified metabolites, O-acetyl-L-serine, Adenine, Creatine, Hydroxyisocaproic acid, deoxyuridine, Creatinine, Allantoin and Histidine exhibited AUC values greater than 0.7, indicating good to moderate abilities in discriminating between fertile men and teratozoospermic infertile patients. Then, by combining the above-mentioned metabolites, a cumulative ROC curve analysis was obtained, showing an AUC of 0.881 and a sensitivity and specificity of 1 and 0.678, respectively (Figure 6I).

### 3.4 Pathway analysis

Table 2 lists the corresponding pathways that each identified biomarker was involved in.

**FIGURE 7:**
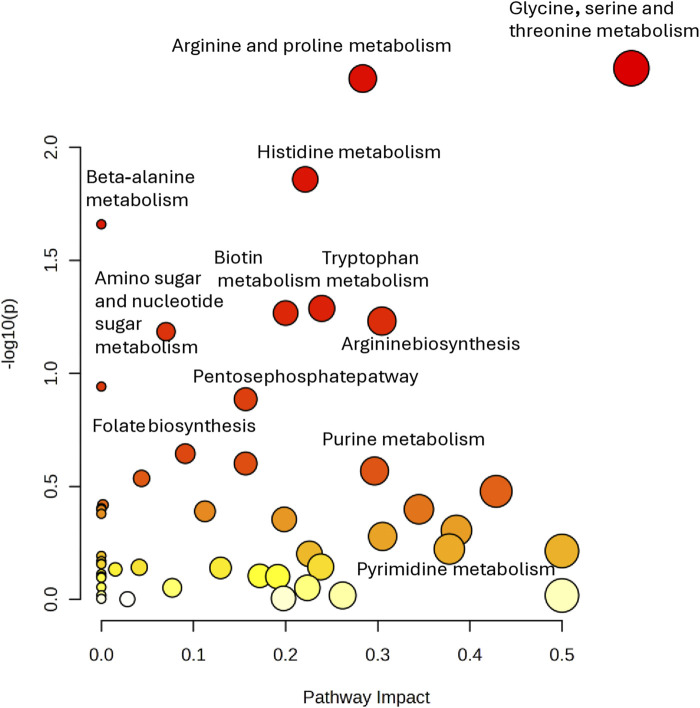
Pathway analysis performed on the 14 significantly altered metabolites (*p* < 0.05) in TZ vs. NZ patients. All matched pathways are plotted according to–log (*p*-value) and pathway impact score. Color gradient and circle size represent the significance of the pathway ranked by p-value (darker colors represent more significant changes of metabolites in the corresponding pathway) and pathway impact score (the larger the circle, the higher the impact score), respectively.

Then, in order to analyze the most relevant metabolic pathway involved in teratozoospermia, pathway analysis was performed using Metaboanalyst. As shown in the scatter plot in Figure 7, the pathways that were found to be potentially affected in our dataset between fertile and TZ men were Arginine and proline metabolism (impact score = 0.575, p-value = 0.004), Glycine, serine and threonine metabolism (impact score = 0.284, p-value = 0.005) and Histidine metabolism (impact score = 0.221, p-value = 0.01). The criteria were set as impact score >0.1 and p-value <0.05 (Figure 7).

## 4 Discussion

This investigation, using an untargeted HPLC-MS-based metabolomic approach, directly compared for the first time the metabolomic profile of human SP from TZ and NZ (fertile) subjects, in order to better understand the alteration of metabolic pathways associated with teratozoospermia. The reported results provided an extensive characterization of the SP metabolome in TZ individuals, which help to distinguish them from individuals with normal sperm morphology, offering new insights for in-vestigating into the pathogenesis of this condition.

Until now, only one MS-based investigation directly compared the metabolomic profile of SP from TZ infertile patients and fertile men by using a targeted amino acids LC–MS/MS (Hosseini et al., 2023). The present investigation provides an untargeted approach aimed at discovering and expanding the range of detectable metabolites in SP samples. Therefore, this strategy offers the great potential for identifying novel metabolic alterations and uncovering uncharacterized biomarkers specifically associated to teratozoospermia.

In the following sections, the most relevant metabolites and metabolic pathways that exhibit significant alterations in TZ patients will be discussed.

A schematic summary of the main metabolic alterations observed in TZ patients is provided in Supplementary Scheme S1, to visually illustrate the key findings discussed.

### 4.1 Changes in creatine and creatinine levels involved in arginine and proline metabolism

In accordance with our data, two previous investigations reported a dysregulation in arginine and proline metabolism associated to teratozoospermia (Mehrparvar et al., 2020; Hosseini et al., 2023).

In our study, the alteration of arginine and proline metabolism was evidenced by a significant reduction of creatine levels in TZ patients compared to NZ men (Table 2; Figure 3).

Creatine is a key component of energy metabolism, essential for normal growth, development, and overall health (Ostojic and Forbes, 2022).

Creatine acts as a vital intermediary in energy transfer, involved in the recycling of ATP, the primary energy source for cellular processes. Consequently, creatine is concentrated in organs with high energy demands, including skeletal muscle, the brain, liver, kidneys, and testes (McCall and Persky, 2007).

In sperm cells creatine plays a crucial role in the phosphocreatine shuttle, a vital metabolic pathway for energy transfer, by transporting energy (ATP) from the site of ATP synthesis (mitochondria) to the site of its utilization, the flagellum involved in the activation of movement (Ostojic and Forbes, 2022). In addition, Creatine kinase, an enzyme that catalyzes the phosphorylation of Creatine to phosphocreatine, facilitates the regeneration of energy within this shuttle (Banihani and Abu-Alhayjaa, 2016). Notably, it has been demonstrated that inactivating creatine kinase can disrupt sperm motility. As a result, evaluating creatine–phosphocreatine shuttle biomarkers is commonly used to assess sperm health (Nasrallah et al., 2020).

Srivastava et al. (1984) conducted one of the earliest studies on the relationship between seminal creatine concentration and sperm viability. They found that creatine levels were typically higher in fertile men compared to infertile ones, indicating the significant role of creatine in sperm quality (Srivastava et al., 1984). Recent studies also found that low semen creatine levels correlate with reduced sperm motility, while high creatine kinase activity is linked to poor sperm quality (Nasrallah et al., 2020). Overall, these findings imply that restoring normal creatine metabolism in sperm could improve sperm quality in men with fertility issues. Several *in vitro* and animal studies have explored the use of exogenous creatine to improve sperm quality. In fact, adding creatine to insemination media has been shown to enhance sperm fertilizing capacity, during *in vitro* fertilization (Ostojic et al., 2022). Creatine also improved sperm capacitation by increasing ATP levels when included in the fertilization medium (Umehara et al., 2018). While these findings are preliminary, they provide evidence that creatine supplements could enhance sperm fertilization ability, highlighting the need for further research on dietary creatine’s effects in male infertility contexts.

In light of these reported considerations, the reduction of creatine levels in TZ patients found in our study might suggest a link with the alteration of the sperm morphology in these patients. This is supported by the significant positive correlation found between creatine levels and sperm morphology parameters (Figure 5). This could suggest the potential use of creatine as a nutritional supplement to enhance sperm quality in TZ men and could lead to the development of possible targeted therapeutic interventions for treatment and management of teratozoospermia.

In this study, Creatinine levels were significantly decreased in TZ patients compared to NZ men (Table 2; Figure 3) and these results were supported by a previous study by Mumcu et al. in which the Authors demonstrated a decrease of creatinine in oligoasthenoteratozoospermic (OAT) patients (Mumcu et al., 2020). Creatinine is a non-protein nitrogenous compounds (NPNs), which is generated from the metabolism of Creatine, involved in high cellular energy metabolism (Wyss and Kaddurah-Daouk, 2000). Its generation and excretion are tightly regulated, reflecting muscle mass, renal function and it is used for estimation of glomerular filtration rate (eGFR) (Wallimann et al., 2011). While alterations in creatinine levels have been commonly used as markers of renal dysfunction (Kellum et al., 2021), its specific role in sperm morphology and function remains underexplored. The observed creatinine reduction in TZ patients could indicate a dysregulation in creatine metabolism, potentially influencing sperm morphology through energy deficits or oxidative stress imbalances. Spermatozoa rely heavily on ATP for processes such as motility, acrosome reaction, and fertilization (du Plessis et al., 2015). Impaired energy metabolism, as indicated by altered creatinine levels, might compromise these functions, resulting in abnormal sperm morphology. Additionally, in a very recent study (Kitlinski et al., 2024) researchers investigated the association between male infertility and renal function. Using creatinine-based measures to estimate eGFR, they found that infertile men were more likely to exhibit decreased eGFR, indicating renal dysfunction. This suggested that infertile men have a higher risk of developing renal disease compared to fertile men, underlining the need of routine kidney function evaluation in this population (Kitlinski et al., 2024).

Further investigations are warranted to elucidate the mechanisms linking creatinine metabolism with teratozoospermia. Targeted studies on metabolic pathways, such as arginine and proline metabolism could identify biomarkers for early diagnosis and provide deeper insights into the pathophysiology of teratozoospermia and related male infertility disorders.

### 4.2 Alteration of histidine metabolism

Another significant pathway that was found to be altered in TZ was histidine metabolism (Figure 7), proved by the lower levels of histidine in the SP of NZ patients compared to TZ men (Figure 4).

The dysregulation of histidine metabolism was evidenced in a very recent study where histidine was found to be upregulated in a rat model of male infertility, compared to control (Shen et al., 2024).

Furthermore, increased levels of histidine were also reported in a previous study by Zhang et al. (2015) in asthenozoospermic patients in comparison to healthy controls (Zhang et al., 2015). Additionally, Xu et al. (2020) demonstrated increased levels of histidine in infertile patients with alteration of sperm count, motility and morphology (OAT), which supports our results. Histidine, an essential amino acid, was found to be a potential inflammatory metabolite linked to oxidative stress, which could explain its elevated levels observed in infertile TZ patients. In fact, as above discussed, in general male infertility is frequently associated with the increased oxidative stress which can impair sperm function (Hussain et al., 2023) and in particular, abnormal spermatozoa morphology has been correlated with an increase in sperm damage markers, including DNA fragmentation or overproduction of ROS (Candela et al., 2021; Agarwal et al., 2014; Ammar et al., 2020).

It is tempting to speculate that the involvement of histidine in oxidative stress could arise from its role as a precursor of bioactive compounds, such as histamine. In fact, one of the pathways of HIS metabolism includes the decarboxylation of L-histidine by L-Histidine decarboxylase (HDC) to generate histamine (Huang et al., 2018), a well-known proinflammatory factor that prompts immune cells to produce inflammatory mediators and cytokines (Branco et al., 2018). Interestingly, the expression of HDC has been found in spermatidis and spermatozoa of male mice which may indicate histamine production in the acrosomes (Safina et al., 2002), suggesting a possible involvement of histamine in the mouse reproductive system. Additionally, HDC expression has also been observed in different compartments of human testis both in fertile and infertile men, suggesting the existence of a histaminergic system in the human testis (Albrecht et al., 2005).

Despite these observations, the precise function of histamine in the male reproductive system remains unclear and no investigations to date have reported a difference in the levels of histamine between fertile and infertile men.

However, it is plausible to suggest that the elevated histidine levels observed in TZ patients in our study may be linked to an increase in the histamine level which plays a critical role in inflammatory responses by enhancing the release of histamine and other pro-inflammatory mediators, leading also to increased ROS production. The increase in histidine in infertile men, particularly those with teratozoospermia, may reflect the body’s attempt to regulate inflammation and repair tissue damage. This might be also part of the body’s defense mechanism against ROS, which are commonly elevated in male infertility and teratozoospermia.

In the future, it would be of great interest to evaluate the level of ROS and other oxidative stress biomarkers in our study cohort in order to validate these considerations. Additionally, further studies are needed to clarify the specific role of histamine and histidine in male infertility, especially in the case of teratozoospermia, given also the presence of a statistically significant negative correlation between sperm morphology and histidine observed in our study (Figure 5).

### 4.3 Changes in O-acetyl-L-serine levels involved in Cysteine and methionine metabolism

Among all the significantly different metabolites found in our investigation, O-acetyl-L-serine, belonging to Cysteine and methionine metabolism, exhibited the most significant p-value and the highest VIP score (Figure 1; Table 2). It was found to be increased in TZ compared to NZ (Figure 4) and also showed a significant negative correlation with sperm morphology (Figure 5) and a good discriminating ability, in terms of AUC, in differentiating TZ from NZ (Figure 6).

The increased levels of O-acetyl-L-serine were previously reported in the serum of azoospermic infertile patients compared to healthy controls, supporting our data (Zhang et al., 2017). This result needs further investigation about the role of O-acetyl-L-serine in teratozoospermia.

### 4.4 Alterations in purine and pyrimidine metabolisms

Purine and pyrimidine metabolisms were other additional metabolic pathways altered in our investigation, confirmed by the dysregulation of deoxyuridine (Pyrimidine metabolism), Adenine, Allantoin and dATP (Purine metabolism) (Table 2).

Purines and pyrimidines are key biomolecules essential for energy storage in the form of ATP and GTP. They are crucial for genetic information transfer, playing a role in cell signaling through molecules like cAMP and cGMP, and also functioning as cofactors (such as NADH, NADPH, and coenzyme A) for various enzymes (Zeng et al., 2018).

Purines were found to be involved in germ cell development, ovarian function, and pregnancy outcomes (Rong et al., 2024). It was shown that purinergic signaling, activated by extracellular ATP, plays a role in germ cell maturation at various developmental stages. Notably, purinoceptors are present in all cell types within the seminiferous tubule (Mundt et al., 2022). Studies showed that removing these receptors in male mice can result in infertility (Belardin et al., 2022).

Alteration of purine and pyrimidine metabolism in infertile men has been reported in some previous studies on SP, which showed dysregulation in the levels of Xanthine, Adenine, Cytosine, Uridine, Uric acid, Cytidine, Inosine, Guanosine, Guanine (Chen et al., 2020; Lazzarino et al., 2018) in different categories of infertile men. Among these, for example, Uridine has also been identified as an oxidative stress biomarker of male infertility in urinary and seminal plasma samples (Maher et al., 2008; Shen et al., 2013).

Modifications in purine and pyrimidine, together with other alterations including double-strand breaks, formation of free-base sites, DNA fragmentation, and DNA crosslinking, may arise from DNA damage in sperm (Barati et al., 2020), which is caused by high levels of oxidative stress and ROS, often associated with teratozoospermia sperm (Ammar et al., 2020). In cases of severe oxidative DNA damage, sperm cells often degenerate and are reabsorbed during spermatogenesis and maturation in the epididymis, leading to reduced sperm quality in affected individuals.

The change in the levels of some metabolites belonging to purine and pyrimidine metabolism in TZ compared to NZ in the present investigation could reflect increased oxidative stress during spermatogenesis in these types of patients, eventually causing infertility. However, specific molecular mechanisms involved still need further studies.

## 5 Conclusion

Our findings provide valuable insights into the underlying mechanisms of teratozoospermia by identifying specific metabolites and metabolic pathways that affect this subcategory of male infertility conditions. Understanding of molecular mechanisms about targeting central metabolic pathway always plays a key role in the discovery of drug targets for optimal therapies.

Significant changes were observed in Creatine, Histidine, Adenine, Allantoin and Deoxyuridine levels, implicating dysregulations in Arginine and Proline metabolism, Histidine metabolism, and Purine/Pyrimidine metabolism. Diagnostic potential of all metabolites was evaluated through ROC curve analysis providing predictive markers of clinical outcomes. Specifically, O-acetyl-L-serine, Adenine, Creatine, Hydroxyisocaproic acid, Deoxyuridine, Creatinine, Allantoin and Histidine showed robust discriminatory power (AUC >0.7) between NZ fertile and TZ infertile men (Figures 6A–H). Additionally, considering that multiple biomarkers pattern could enhance the accuracy of prediction models than any single biomarker alone, we performed the ROC curve analysis from the combinations of the above mentioned top eight biomarkers (Figure 6I) showed the highest AUC = 0.881, with sensitivity and specificity equal to 100% and 67.8%, respectively. The ROC model achieved improved diagnostic performance, demonstrating that multi-markers tools could better provide a global metabolic snapshot of infertility conditions improving clinical management of disease. It is important to note that while significant correlations were identified between specific metabolites and sperm morphology, correlation does not necessarily imply causation. Further studies are needed to determine whether these metabolites play a direct role in the pathophysiology of teratozoospermia or are merely associated with the condition.

Using a HPLC-MS based untargeted approach, metabolomics profiling of SP from NZ fertile and TZ infertile men was performed and specific SP molecular signatures able to differentiate patients were generated. Specifically, 5 significantly reduced and 9 significantly elevated metabolites in TZ patients compared to NZ men were identified. Interestingly, these candidate differential metabolites, impact metabolic pathways and biological processes involved in inflammation, oxidative stress and sperm DNA damage. Indeed, our results revealed interesting interconnections of metabolic networks suggesting that, the elevated levels of metabolites able to exacerbate inflammation observed in TZ patients, may also contribute to oxidative stress which can be responsible for harmful effects on various sperm structures and significant DNA damage. Thus, we also hypothesized that the statistically significant difference in progressive motility between the two analyzed groups, could be attributed to a potential association between sperm structures damages and reduced motility observed in our TZ cohort (Table 1).

Such results are in accordance with previous investigations which reported that morphologically abnormal spermatozoa by producing increased levels of ROS, could promote other sperm structure damage and additional aspects of sperm dysfunction such as sperm motility and DNA damage during spermatogenesis (Atmoko et al., 2024; Cao et al., 2017) which could also negatively impact early embryo development, resulting in spontaneous abortions (Raj et al., 2021).

In conclusion, these findings suggest that our pilot metabolomics approach might be strongly predictive for teratozoospermia risk classification, with great potential as useful tool for decision-making in clinical medicine. The results revealed distinct metabolic signatures between the two groups, suggesting potential metabolic alterations associated with sperm morphology abnormalities. However, while these findings provide valuable insights into the metabolic differences in TZ individuals, several limitations must be acknowledged before drawing definitive biological conclusions.

Firstly, the lack of external validation limits the generalizability of the findings. Although the study identified a set of metabolites significantly associated with teratozoospermia, further validation in independent, larger cohorts is necessary to confirm the robustness and clinical relevance of these metabolic markers. Secondly, the possibility of artifactual metabolic changes due to sample handling and processing should be considered. Despite efforts to standardize sample collection and processing, and microscopic verification to ensure the absence of spermatozoa, minor disruption of sperm cells during high-speed centrifugation may have released intracellular metabolites into the seminal plasma fraction. This potential contamination could affect metabolite profiles and bias pathway enrichment analyses. Therefore, pathway interpretations should be considered exploratory and confirmed by further functional validation in independent cohorts. Future studies should aim to minimize such contamination and validate findings in independent cohorts. Overall, while this study provides a comprehensive metabolomic characterization of SP in TZ individuals, it remains exploratory. Future research should focus on independent validation, longitudinal studies, and mechanistic investigations to better understand the role of these metabolic changes in male infertility. Additionally, integrating functional sperm analyses and proteomic data could enhance our understanding of the metabolic underpinnings of sperm morphology abnormalities.

Thus, predictive ability and diagnostic power of the established metabolic signatures need further validation stage on larger sample sizes to clinical translation over time. If validated in diverse cohorts, this tool could be useful to identify functional biomarkers and metabolomics pathways alterations as early indicators of sperm dysfunction and damage during pre-disease states, but also for monitoring progression of disease and enabling personalized patient management with precision treatment.

## Data Availability

The original contributions presented in the study are included in the article/Supplementary Material, further inquiries can be directed to the corresponding author.
