# Supplementary material for: Untargeted metabolomics fingerprints in seminal plasma of patients with abnormal sperm morphology using high-performance liquid chromatography and mass spectrometry
Source: Front Mol Biosci. 2025 Jun 17;12:1578998. doi: 10.3389/fmolb.2025.1578998 (PMC12208838; doi:10.3389/fmolb.2025.1578998)
Supplement: Supplementary file 1 [file Supplementaryfile1.docx]

Supplementary Material

**Table S1.** Smoking and alcohol status, and Use of Other Substances in Teratozoospermic patients (n=15) vs Normozoospermic men (n=20).

| **Patients’s characteristics** | | **Normozoospermic (NZ)** | **Teratozoospermic (TZ)** |
| --- | --- | --- | --- |
|  | Age (years) | 26,3 ± 6,5 | 33,7 ± 7,6 |
| Smoking status | Non-smokers (number) | 10 | 4 |
|  | Smokers (number) | 10 | 11 |
| Alcohol status | Non-alcoholics (number) | 9 | 3 |
|  | Alcoholics (number) | 11 | 12 |
| Use of other substances |  | None | None |

Data are expressed as numbers or mean ± standard deviation.

**Table S2**. Metabolites used in the analysis.

| **Spearman's Correlations** | | | | | |
| --- | --- | --- | --- | --- | --- |
| **Variable** | |  | | **Morphology** | |
| 1. Morphology |  | Spearman's rho |  | — |  |
|  |  | p-value |  | — |  |
| 2. 1-Methyladenosine |  | Spearman's rho |  | 0.254 |  |
|  |  | p-value |  | 0.141 |  |
| 3. 2-Aminooctanoic acid |  | Spearman's rho |  | 0.089 |  |
|  |  | p-value |  | 0.611 |  |
| 4. 2-Isopropylmalic acid |  | Spearman's rho |  | -0.313 |  |
|  |  | p-value |  | 0.067 |  |
| 5. 2-keto-isovalerate |  | Spearman's rho |  | -0.117 |  |
|  |  | p-value |  | 0.504 |  |
| 6. 2-oxo-4-methylthiobutanoate |  | Spearman's rho |  | -0.050 |  |
|  |  | p-value |  | 0.777 |  |
| 7. 4-Pyridoxic acid |  | Spearman's rho |  | -0.097 |  |
|  |  | p-value |  | 0.581 |  |
| 8. 5-methoxytryptophan |  | Spearman's rho |  | -0.043 |  |
|  |  | p-value |  | 0.805 |  |
| 9. 5-methyl-THF |  | Spearman's rho |  | -0.154 |  |
|  |  | p-value |  | 0.378 |  |
| 10. 6-phospho-D-gluconate |  | Spearman's rho |  | -0.418 | * |
|  |  | p-value |  | 0.013 |  |
| 11. 7_8-dihydrofolate |  | Spearman's rho |  | -0.088 |  |
|  |  | p-value |  | 0.615 |  |
| 12. 7-methylguanosine |  | Spearman's rho |  | 0.025 |  |
|  |  | p-value |  | 0.887 |  |
| 13. acadesine |  | Spearman's rho |  | 0.230 |  |
|  |  | p-value |  | 0.184 |  |
| 14. Acetylcarnitine |  | Spearman's rho |  | 0.216 |  |
|  |  | p-value |  | 0.212 |  |
| 15. Acetyllysine |  | Spearman's rho |  | -0.213 |  |
|  |  | p-value |  | 0.218 |  |
| 16. aconitate |  | Spearman's rho |  | 0.374 | * |
|  |  | p-value |  | 0.027 |  |
| 17. adenine |  | Spearman's rho |  | 0.256 |  |
|  |  | p-value |  | 0.138 |  |
| 18. adenosine |  | Spearman's rho |  | 0.242 |  |
|  |  | p-value |  | 0.162 |  |
| 19. ADP-D-glucose |  | Spearman's rho |  | 0.031 |  |
|  |  | p-value |  | 0.861 |  |
| 20. a-ketoglutarate |  | Spearman's rho |  | 0.315 |  |
|  |  | p-value |  | 0.066 |  |
| 21. allantoin |  | Spearman's rho |  | -0.367 | * |
|  |  | p-value |  | 0.030 |  |
| 22. Aminoadipic acid |  | Spearman's rho |  | 0.162 |  |
|  |  | p-value |  | 0.352 |  |
| 23. aminoimidazole carboxamide ribonucleotide |  | Spearman's rho |  | -0.096 |  |
|  |  | p-value |  | 0.582 |  |
| 24. anthranilate |  | Spearman's rho |  | -0.034 |  |
|  |  | p-value |  | 0.847 |  |
| 25. arginine |  | Spearman's rho |  | -0.287 |  |
|  |  | p-value |  | 0.095 |  |
| 26. asparagine |  | Spearman's rho |  | 0.229 |  |
|  |  | p-value |  | 0.185 |  |
| 27. aspartate |  | Spearman's rho |  | -0.366 | * |
|  |  | p-value |  | 0.031 |  |
| 28. ATP |  | Spearman's rho |  | -0.131 |  |
|  |  | p-value |  | 0.454 |  |
| 29. Atrolactic acid |  | Spearman's rho |  | -0.088 |  |
|  |  | p-value |  | 0.617 |  |
| 30. biotin |  | Spearman's rho |  | 0.201 |  |
|  |  | p-value |  | 0.248 |  |
| 31. carnitine |  | Spearman's rho |  | -0.002 |  |
|  |  | p-value |  | 0.990 |  |
| 32. CDP-ethanolamine |  | Spearman's rho |  | 0.249 |  |
|  |  | p-value |  | 0.148 |  |
| 33. cholesteryl sulfate |  | Spearman's rho |  | -0.103 |  |
|  |  | p-value |  | 0.557 |  |
| 34. Cholic acid |  | Spearman's rho |  | -0.193 |  |
|  |  | p-value |  | 0.266 |  |
| 35. choline |  | Spearman's rho |  | 0.143 |  |
|  |  | p-value |  | 0.412 |  |
| 36. Citraconic acid |  | Spearman's rho |  | 0.046 |  |
|  |  | p-value |  | 0.791 |  |
| 37. citrulline |  | Spearman's rho |  | -0.124 |  |
|  |  | p-value |  | 0.477 |  |
| 38. CMP |  | Spearman's rho |  | 0.063 |  |
|  |  | p-value |  | 0.721 |  |
| 39. creatine |  | Spearman's rho |  | 0.380 | * |
|  |  | p-value |  | 0.024 |  |
| 40. Creatinine |  | Spearman's rho |  | 0.180 |  |
|  |  | p-value |  | 0.300 |  |
| 41. CTP |  | Spearman's rho |  | 0.142 |  |
|  |  | p-value |  | 0.414 |  |
| 42. cytidine |  | Spearman's rho |  | 0.107 |  |
|  |  | p-value |  | 0.541 |  |
| 43. cytosine |  | Spearman's rho |  | -0.053 |  |
|  |  | p-value |  | 0.762 |  |
| 44. dAMP |  | Spearman's rho |  | -0.002 |  |
|  |  | p-value |  | 0.993 |  |
| 45. dATP |  | Spearman's rho |  | -0.460 | ** |
|  |  | p-value |  | 0.005 |  |
| 46. dCMP |  | Spearman's rho |  | -0.293 |  |
|  |  | p-value |  | 0.087 |  |
| 47. deoxyadenosine |  | Spearman's rho |  | 0.273 |  |
|  |  | p-value |  | 0.112 |  |
| 48. Deoxycholic acid |  | Spearman's rho |  | -0.068 |  |
|  |  | p-value |  | 0.699 |  |
| 49. deoxyinosine |  | Spearman's rho |  | -0.033 |  |
|  |  | p-value |  | 0.851 |  |
| 50. deoxyuridine |  | Spearman's rho |  | 0.083 |  |
|  |  | p-value |  | 0.635 |  |
| 51. D-glucarate |  | Spearman's rho |  | 0.248 |  |
|  |  | p-value |  | 0.151 |  |
| 52. D-gluconate |  | Spearman's rho |  | 0.057 |  |
|  |  | p-value |  | 0.747 |  |
| 53. D-glucono-Î´-lactone-6-phosphate |  | Spearman's rho |  | -0.163 |  |
|  |  | p-value |  | 0.350 |  |
| 54. D-glucosamine-6-phosphate |  | Spearman's rho |  | 0.500 | ** |
|  |  | p-value |  | 0.002 |  |
| 55. dGMP |  | Spearman's rho |  | 0.115 |  |
|  |  | p-value |  | 0.511 |  |
| 56. Diiodothyronine |  | Spearman's rho |  | -0.093 |  |
|  |  | p-value |  | 0.597 |  |
| 57. dimethylglycine |  | Spearman's rho |  | 0.030 |  |
|  |  | p-value |  | 0.864 |  |
| 58. DL-Pipecolic acid |  | Spearman's rho |  | -0.204 |  |
|  |  | p-value |  | 0.240 |  |
| 59. dUMP |  | Spearman's rho |  | -0.023 |  |
|  |  | p-value |  | 0.898 |  |
| 60. FAD |  | Spearman's rho |  | 0.062 |  |
|  |  | p-value |  | 0.724 |  |
| 61. folate |  | Spearman's rho |  | -0.279 |  |
|  |  | p-value |  | 0.105 |  |
| 62. glucono-Î´-lactone |  | Spearman's rho |  | -0.410 | * |
|  |  | p-value |  | 0.014 |  |
| 63. glucosamine |  | Spearman's rho |  | -0.230 |  |
|  |  | p-value |  | 0.185 |  |
| 64. glucose-6-phosphate |  | Spearman's rho |  | -0.203 |  |
|  |  | p-value |  | 0.242 |  |
| 65. glutamine |  | Spearman's rho |  | -0.088 |  |
|  |  | p-value |  | 0.616 |  |
| 66. Glycerophosphocholine |  | Spearman's rho |  | -0.098 |  |
|  |  | p-value |  | 0.575 |  |
| 67. glycine |  | Spearman's rho |  | 0.193 |  |
|  |  | p-value |  | 0.266 |  |
| 68. GTP |  | Spearman's rho |  | 0.101 |  |
|  |  | p-value |  | 0.565 |  |
| 69. Guanidoacetic acid |  | Spearman's rho |  | -0.070 |  |
|  |  | p-value |  | 0.687 |  |
| 70. guanine |  | Spearman's rho |  | 0.115 |  |
|  |  | p-value |  | 0.509 |  |
| 71. guanosine |  | Spearman's rho |  | 0.335 | * |
|  |  | p-value |  | 0.049 |  |
| 72. guanosine 5--diphosphate-3--diphosphate |  | Spearman's rho |  | 0.056 |  |
|  |  | p-value |  | 0.751 |  |
| 73. histidine |  | Spearman's rho |  | -0.408 | * |
|  |  | p-value |  | 0.015 |  |
| 74. homocysteic acid |  | Spearman's rho |  | -0.273 |  |
|  |  | p-value |  | 0.112 |  |
| 75. Hydroxyisocaproic acid |  | Spearman's rho |  | 0.358 | * |
|  |  | p-value |  | 0.035 |  |
| 76. Hydroxyphenylacetic acid |  | Spearman's rho |  | 0.100 |  |
|  |  | p-value |  | 0.567 |  |
| 77. hydroxyphenylpyruvate |  | Spearman's rho |  | -0.161 |  |
|  |  | p-value |  | 0.355 |  |
| 78. hypoxanthine |  | Spearman's rho |  | 0.128 |  |
|  |  | p-value |  | 0.464 |  |
| 79. Imidazoleacetic acid |  | Spearman's rho |  | 0.285 |  |
|  |  | p-value |  | 0.098 |  |
| 80. IMP |  | Spearman's rho |  | 4.328×10^-4^ |  |
|  |  | p-value |  | 0.998 |  |
| 81. indole |  | Spearman's rho |  | -0.304 |  |
|  |  | p-value |  | 0.076 |  |
| 82. Indole-3-carboxylic acid |  | Spearman's rho |  | -0.070 |  |
|  |  | p-value |  | 0.691 |  |
| 83. Indoleacrylic acid |  | Spearman's rho |  | -0.310 |  |
|  |  | p-value |  | 0.070 |  |
| 84. inosine |  | Spearman's rho |  | 0.183 |  |
|  |  | p-value |  | 0.294 |  |
| 85. isocitrate |  | Spearman's rho |  | 0.236 |  |
|  |  | p-value |  | 0.172 |  |
| 86. Kynurenic acid |  | Spearman's rho |  | 0.126 |  |
|  |  | p-value |  | 0.470 |  |
| 87. Kynurenine |  | Spearman's rho |  | 0.032 |  |
|  |  | p-value |  | 0.853 |  |
| 88. lactate |  | Spearman's rho |  | 0.269 |  |
|  |  | p-value |  | 0.118 |  |
| 89. L-arginino-succinate |  | Spearman's rho |  | 0.384 | * |
|  |  | p-value |  | 0.023 |  |
| 90. lipoate |  | Spearman's rho |  | -0.119 |  |
|  |  | p-value |  | 0.496 |  |
| 91. lysine |  | Spearman's rho |  | -0.054 |  |
|  |  | p-value |  | 0.758 |  |
| 92. malate |  | Spearman's rho |  | -0.053 |  |
|  |  | p-value |  | 0.762 |  |
| 93. methionine |  | Spearman's rho |  | -0.095 |  |
|  |  | p-value |  | 0.586 |  |
| 94. Methionine sulfoxide |  | Spearman's rho |  | -0.190 |  |
|  |  | p-value |  | 0.275 |  |
| 95. Methylcysteine |  | Spearman's rho |  | 0.231 |  |
|  |  | p-value |  | 0.183 |  |
| 96. methylnicotinamide |  | Spearman's rho |  | 0.168 |  |
|  |  | p-value |  | 0.336 |  |
| 97. myo-inositol |  | Spearman's rho |  | 0.041 |  |
|  |  | p-value |  | 0.815 |  |
| 98. N-acetyl-glucosamine-1/6-phosphate |  | Spearman's rho |  | -0.221 |  |
|  |  | p-value |  | 0.202 |  |
| 99. N-acetyl-glutamate |  | Spearman's rho |  | 0.031 |  |
|  |  | p-value |  | 0.858 |  |
| 100. N-acetyl-glutamine |  | Spearman's rho |  | -0.037 |  |
|  |  | p-value |  | 0.835 |  |
| 101. N-acetyl-L-ornithine |  | Spearman's rho |  | 0.127 |  |
|  |  | p-value |  | 0.466 |  |
| 102. N-Acetylputrescine |  | Spearman's rho |  | 0.207 |  |
|  |  | p-value |  | 0.233 |  |
| 103. N-carbamoyl-L-aspartate |  | Spearman's rho |  | 0.067 |  |
|  |  | p-value |  | 0.704 |  |
| 104. NG-dimethyl-L-arginine |  | Spearman's rho |  | -0.162 |  |
|  |  | p-value |  | 0.354 |  |
| 105. nicotinamide |  | Spearman's rho |  | 0.152 |  |
|  |  | p-value |  | 0.384 |  |
| 106. Nicotinamide ribotide |  | Spearman's rho |  | 0.018 |  |
|  |  | p-value |  | 0.920 |  |
| 107. O-acetyl-L-serine |  | Spearman's rho |  | -0.492 | ** |
|  |  | p-value |  | 0.003 |  |
| 108. Phenylpropiolic acid |  | Spearman's rho |  | -0.060 |  |
|  |  | p-value |  | 0.731 |  |
| 109. phenylpyruvate |  | Spearman's rho |  | -0.196 |  |
|  |  | p-value |  | 0.259 |  |
| 110. Phosphorylcholine |  | Spearman's rho |  | -0.084 |  |
|  |  | p-value |  | 0.630 |  |
| 111. Chorismate |  | Spearman's rho |  | -0.215 |  |
|  |  | p-value |  | 0.214 |  |
| 112. proline |  | Spearman's rho |  | 0.040 |  |
|  |  | p-value |  | 0.818 |  |
| 113. purine |  | Spearman's rho |  | -0.118 |  |
|  |  | p-value |  | 0.500 |  |
| 114. Riboflavin |  | Spearman's rho |  | 0.165 |  |
|  |  | p-value |  | 0.343 |  |
| 115. S-Adenosyl-L-homocysteine |  | Spearman's rho |  | -0.207 |  |
|  |  | p-value |  | 0.233 |  |
| 116. S-Adenosylmethioninamine |  | Spearman's rho |  | 0.137 |  |
|  |  | p-value |  | 0.431 |  |
| 117. S-Adenosyl-L-methionine |  | Spearman's rho |  | 0.053 |  |
|  |  | p-value |  | 0.764 |  |
| 118. Serine |  | Spearman's rho |  | 0.130 |  |
|  |  | p-value |  | 0.458 |  |
| 119. 5--Methylthioadenosine |  | Spearman's rho |  | 0.135 |  |
|  |  | p-value |  | 0.441 |  |
| 120. sn-Glycerol 3-phosphate |  | Spearman's rho |  | -0.117 |  |
|  |  | p-value |  | 0.504 |  |
| 121. S-Ribosyl-L-homocysteine |  | Spearman's rho |  | -0.158 |  |
|  |  | p-value |  | 0.365 |  |
| 122. taurine |  | Spearman's rho |  | 0.243 |  |
|  |  | p-value |  | 0.159 |  |
| 123. Taurodeoxycholate |  | Spearman's rho |  | -8.432×10^-4^ |  |
|  |  | p-value |  | 0.996 |  |
| 124. Thiamine |  | Spearman's rho |  | 0.033 |  |
|  |  | p-value |  | 0.849 |  |
| 125. Threonine |  | Spearman's rho |  | 0.173 |  |
|  |  | p-value |  | 0.322 |  |
| 126. Thymidine |  | Spearman's rho |  | -0.235 |  |
|  |  | p-value |  | 0.174 |  |
| 127. Sucrose |  | Spearman's rho |  | 0.170 |  |
|  |  | p-value |  | 0.330 |  |
| 128. tryptophan |  | Spearman's rho |  | -0.294 |  |
|  |  | p-value |  | 0.086 |  |
| 129. Tyrosine |  | Spearman's rho |  | 0.079 |  |
|  |  | p-value |  | 0.651 |  |
| 130. UDP-glucose |  | Spearman's rho |  | 0.062 |  |
|  |  | p-value |  | 0.722 |  |
| 131. UDP-glucuronate |  | Spearman's rho |  | -0.291 |  |
|  |  | p-value |  | 0.090 |  |
| 132. Uracil |  | Spearman's rho |  | 0.044 |  |
|  |  | p-value |  | 0.803 |  |
| 133. Uric acid |  | Spearman's rho |  | 0.195 |  |
|  |  | p-value |  | 0.261 |  |
| 134. Uridine |  | Spearman's rho |  | 0.637 | *** |
|  |  | p-value |  | < .001 |  |
| 135. Valine |  | Spearman's rho |  | 0.088 |  |
|  |  | p-value |  | 0.615 |  |
| 136. Xanthine |  | Spearman's rho |  | -0.068 |  |
|  |  | p-value |  | 0.698 |  |
| 137. Gluthione disulfide |  | Spearman's rho |  | 0.186 |  |
|  |  | p-value |  | 0.285 |  |
| 138. Glutathione |  | Spearman's rho |  | -0.011 |  |
|  |  | p-value |  | 0.950 |  |
|  | | | | | |
| * p < .05, ** p < .01, *** p < .001 | | | | | |


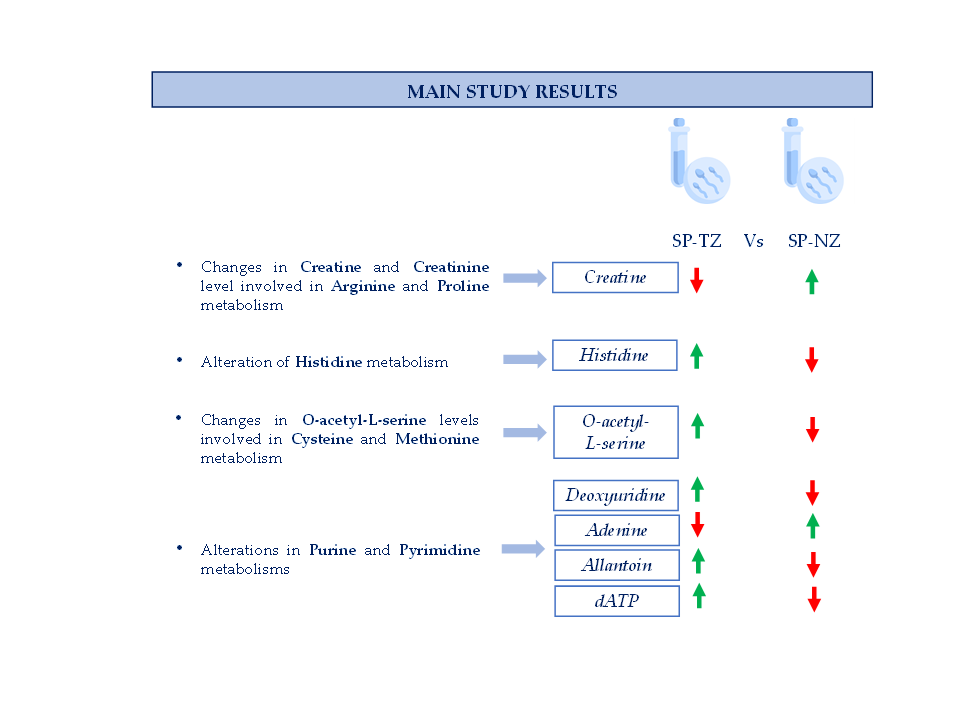


**Supplementary Scheme 1.** Schematic summary of the main metabolic alterations observed in teratozoospermic patients compared to normozoospermic men.
